# Supplementary material for: MINPP1 prevents intracellular accumulation of the chelator inositol hexakisphosphate and is mutated in Pontocerebellar Hypoplasia
Source: Nat Commun. 2020 Nov 30;11:6087. doi: 10.1038/s41467-020-19919-y (PMC7705663; doi:10.1038/s41467-020-19919-y)
Supplement: Supplementary file 1 — Supplementary Information [file 41467_2020_19919_MOESM1_ESM.pdf]

**SUPPLEMENTARY INFORMATION**

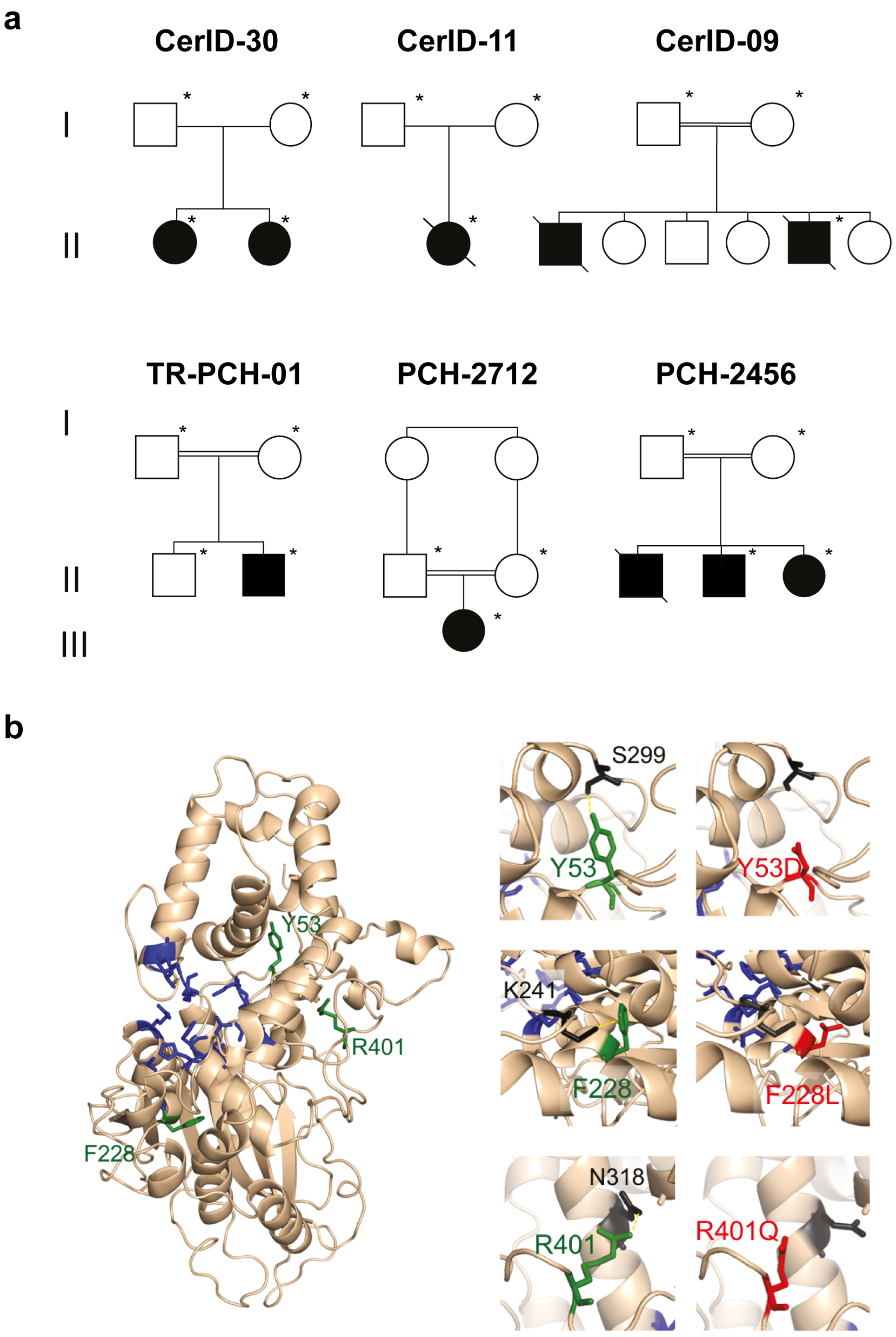


**Supplementary Figure 1. Family pedigrees and *in silico* prediction analysis of the missense *MINPP1* variants. (a**) Pedigrees of families with mutations in the *MINPP1* gene**.** Individuals subjected to Whole Exome Sequencing (WES) or Sanger Sequencing are indicated with asterisk. Additional DNA samples for family CerID-09 could not be collected for segregation analysis. (**b**) Wildtype MINPP1 with amino acids involved in $IP_6$ binding are indicated in blue

and amino acids mutated with missense variants are indicated in green. The Missense3D server predicted that all the three missense variants tested cause structural damage.

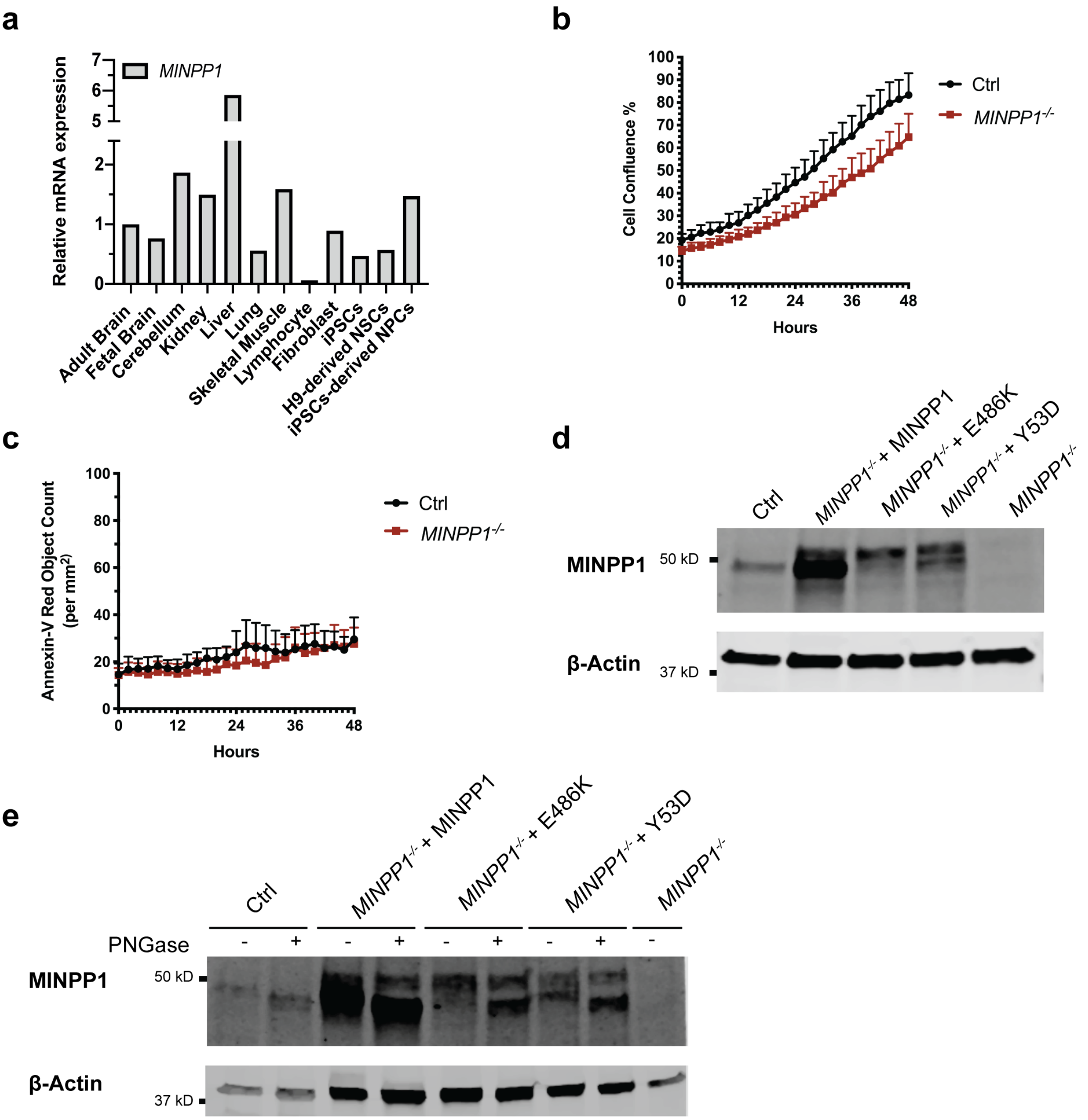


**Supplementary Figure 2: Expression profile of *MINPP1* in different human tissues and protein levels of MINPP1 variants and their corresponding glycosylation status in HEK293 cells. (a)** Quantitative PCR analysis of human *MINPP1* expression, normalized with *ACTB*, in human cells and tissues. Abbreviations used: Neural Stem Cells (NSCs); Neuronal

Progenitor Cells (NPCs). **(b)** Control and *MINPP1*$^{-/-}$ HEK293 were cultured for 48 hours in IncuCyte live-cell analysis system and cell confluence (%) was analysed in IncuCyte Zoom live-cell-imaging software. Graph shows the mean percentage of cell confluence (± s.d.) over time. (n=3, two-tailed student's t-test, not significant). **(c)** Control and *MINPP1*$^{-/-}$ HEK293 were cultured with Annexin-V containing growth media for 48 hours inside the IncuCyte live-cell analysis system, and images were captured with red fluorescence channel to detect Annexin-V positive cells. Graph shows the average ratio (±s.d.) of red cell object count per $mm^2$ (n=3, two-tailed student's t-test, not significant). For **(a-c)**, source data are provided as a source data file. **(d-e)** Western blot data showing the exogenous MINPP1 levels **(d)** and its glycosylated and deglycosylated forms **(e)** in *MINPP1*$^{-/-}$ HEK293 cells transiently transfected with plasmids encoding empty vector, wild type, Y53D or E486K variant MINPP1. MINPP1 is present in control HEK293 cells and absent in *MINPP1*$^{-/-}$ HEK293 cells. β-Actin shown as loading control. Uncropped blots are provided as a source data file and are representative of three and two independent experiments for **(d)** and **(e)**, respectively.

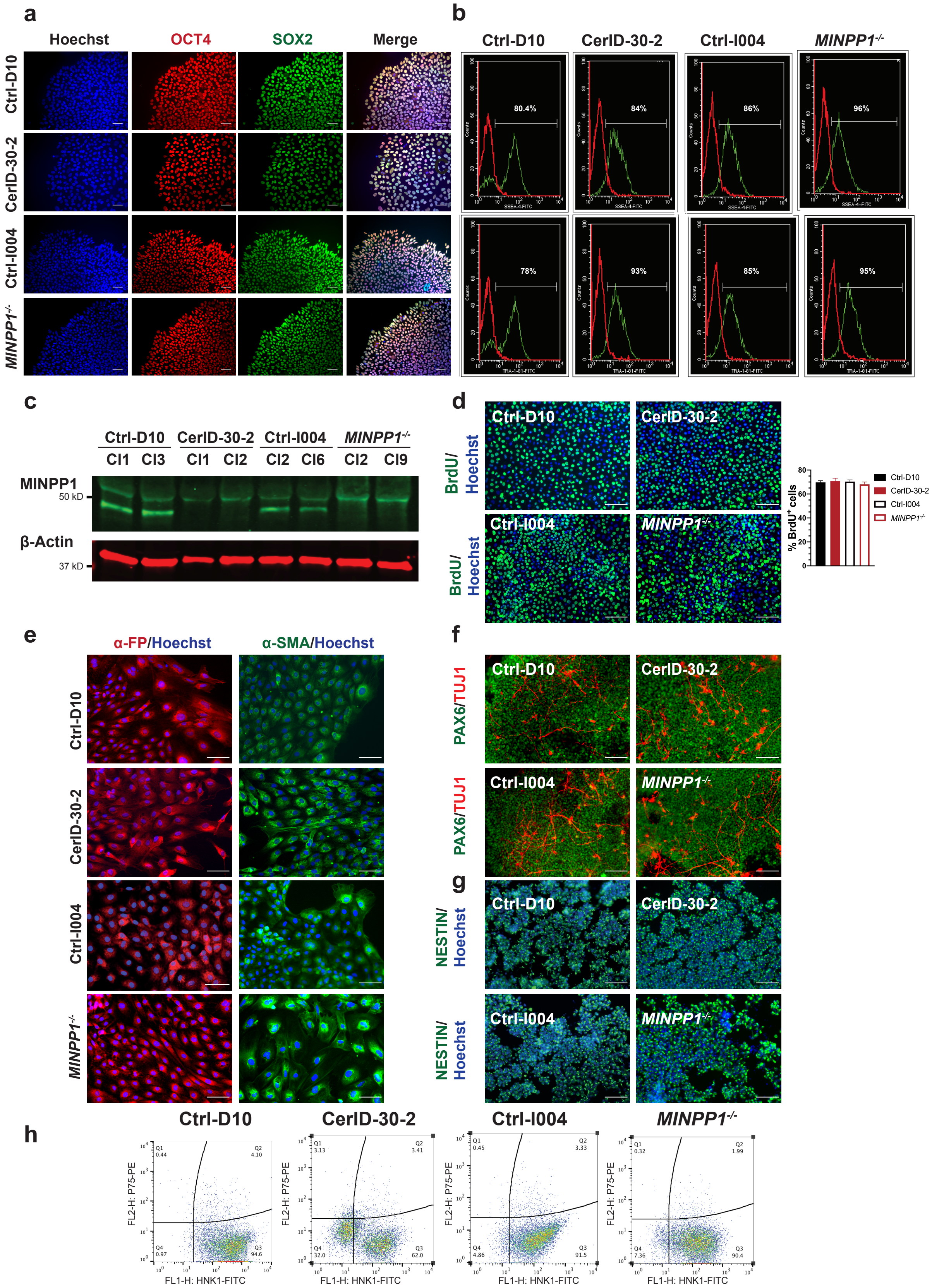
a
Hoechst
OCT4
SOX2
Merge
Ctrl-D10
CerID-30-2
Ctrl-I004
MINPP1-/-
b
Ctrl-D10
CerID-30-2
Ctrl-I004
MINPP1-/-
80.4%
84%
86%
96%
78%
93%
85%
95%
c
Ctrl-D10
CerID-30-2
Ctrl-I004
MINPP1-/-
Cl1 Cl3 Cl1 Cl2 Cl2 Cl6 Cl2 Cl9
MINPP1
50 kD
β-Actin
37 kD
d
BrdU/Hoechst
Ctrl-D10
CerID-30-2
Ctrl-I004
MINPP1-/-
% BrdU+ cells
e
α-FP/Hoechst
α-SMA/Hoechst
f
PAX6/TUJ1
g
NESTIN/Hoechst
h
Ctrl-D10
CerID-30-2
Ctrl-I004
MINPP1-/-
FL2-H: P75-PE
FL1-H: HNK1-FITC

**Supplementary Figure 3. Characterization of patient-derived (CerID-30-2) and *MINPP1$^{-/-}$* iPSCs and their neural derivatives. (a)** Immunofluorescence staining of embryonic stem cell markers OCT4 and SOX2 and **(b)** flow cytometer analysis of pluripotency markers SSEA-4 and TRA-1-81 in control (Ctrl-D10 and Ctrl-I004), patient-derived (CerID-30-2), and *MINPP1$^{-/-}$* iPSCs. Images provided in **(a)** represents one of three independent experiments. **(c)** Western blot data showing the absence of MINPP1 in patient-derived CerID-30-2 and *MINPP1$^{-/-}$* iPSCs. MINPP1 (48 kDa) is present in controls (Ctrl-D10 and Ctrl-I004) iPSC lines (lower band). β-actin is shown as loading control. Uncropped blot is provided as a source data file and is representative of three independent experiments. **(d)** Representative images and quantification data of BrdU$^{+}$ staining assay in control (Ctrl-D10 and Ctrl-I004), patient-derived (CerID-30-2), and *MINPP1$^{-/-}$* iPSCs. (n=3, one way-ANOVA, not significant). The data are presented as mean percentage values ± s.d and source data are provided as a source data file. **(e)** Immunofluorescence staining of endoderm (alpha-Fetoprotein(AFP)) and mesoderm (smooth muscle actin (α-SMA)) markers following embryoid formation from control (Ctrl-D10 and Ctrl-I004), patient-derived (CerID-30-2), and *MINPP1$^{-/-}$* iPSCs. **(f-g)** Representative immunofluorescence images of (**f**) PAX6 /TUJ1 and (**g**) NESTIN for control (Ctrl-D10 and Ctrl-I004), patient-derived (CerID-30-2) and *MINPP1$^{-/-}$* iPSCs during neuronal differentiation at day 10 and day 14, respectively. For **(e-g)**, images are representative of one **(e, g)** and two-independent cell culture experiments **(f)**, respectively. **(h)** Flow cytometer analysis of P75 and HNK1 in control (Ctrl-D10 and Ctrl-I004), patient-derived (CerID-30-2) and *MINPP1$^{-/-}$* neural rosette cultures. All scale bars correspond to 50 μm.

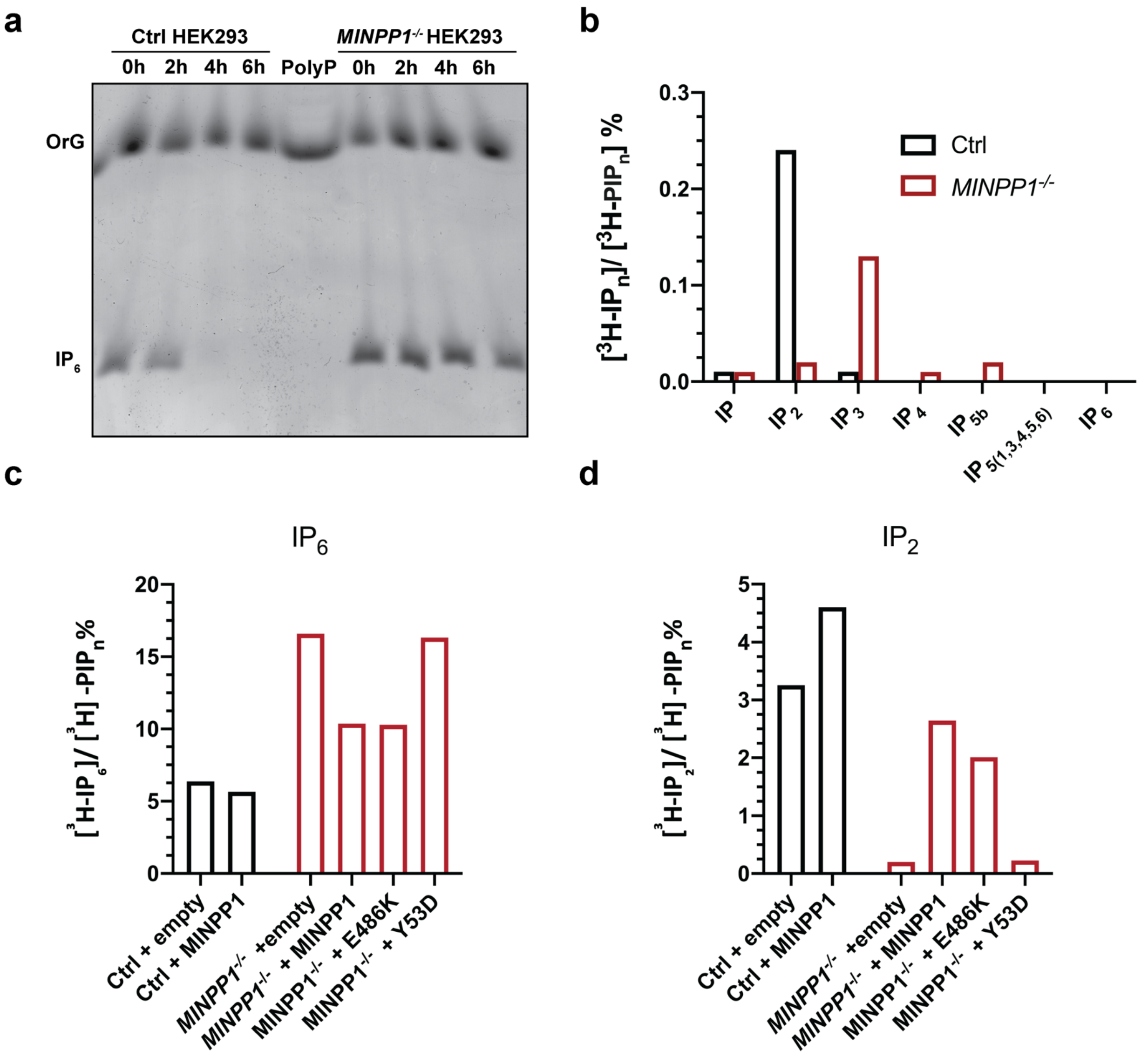


**Supplementary Figure 4. Phytase activity and SAX-HPLC analysis of extracellular and intracellular inositol phosphate levels.** (**a**) MINPP1 phytase activity in conditioned medium of *MINPP1*$^{-/-}$ HEK293 cells upon addition of 4 nmol of $IP_6$ and incubation at 37°C for the time period indicated above. The samples were then mixed with Orange G (OrG) loading dye and resolved by PAGE followed by toluidine blue staining. Gel image representative of two independent experiments. Polyphosphate (PolyP) is shown as size ladder. Uncropped blot is

provided as a source data file. (**b**) SAX-HPLC analysis of inositol phosphate levels in cell culture media of [$^3$H]-inositol labeled control and *MINPP1*$^{-/-}$ HEK293 cells. **(c-d)** SAX-HPLC analysis of $IP_2$ and $IP_6$ levels in control and *MINPP1*$^{-/-}$ HEK293 cells transiently transfected with plasmids encoding empty vector, wildtype, Y53D or E486K variant MINPP1. The data is representative of two independent experiments. [$^3$H]-$IP_n$ levels are presented as percentage of total radioactivity in the inositol-lipid fraction ([$^3$H]-$PIP_n$). Abbreviations used: $IP_n$, inositol phosphates; $PIP_n$, phosphatidyl inositol phosphates. For **(b-d)**, source data are provided as a source data file.

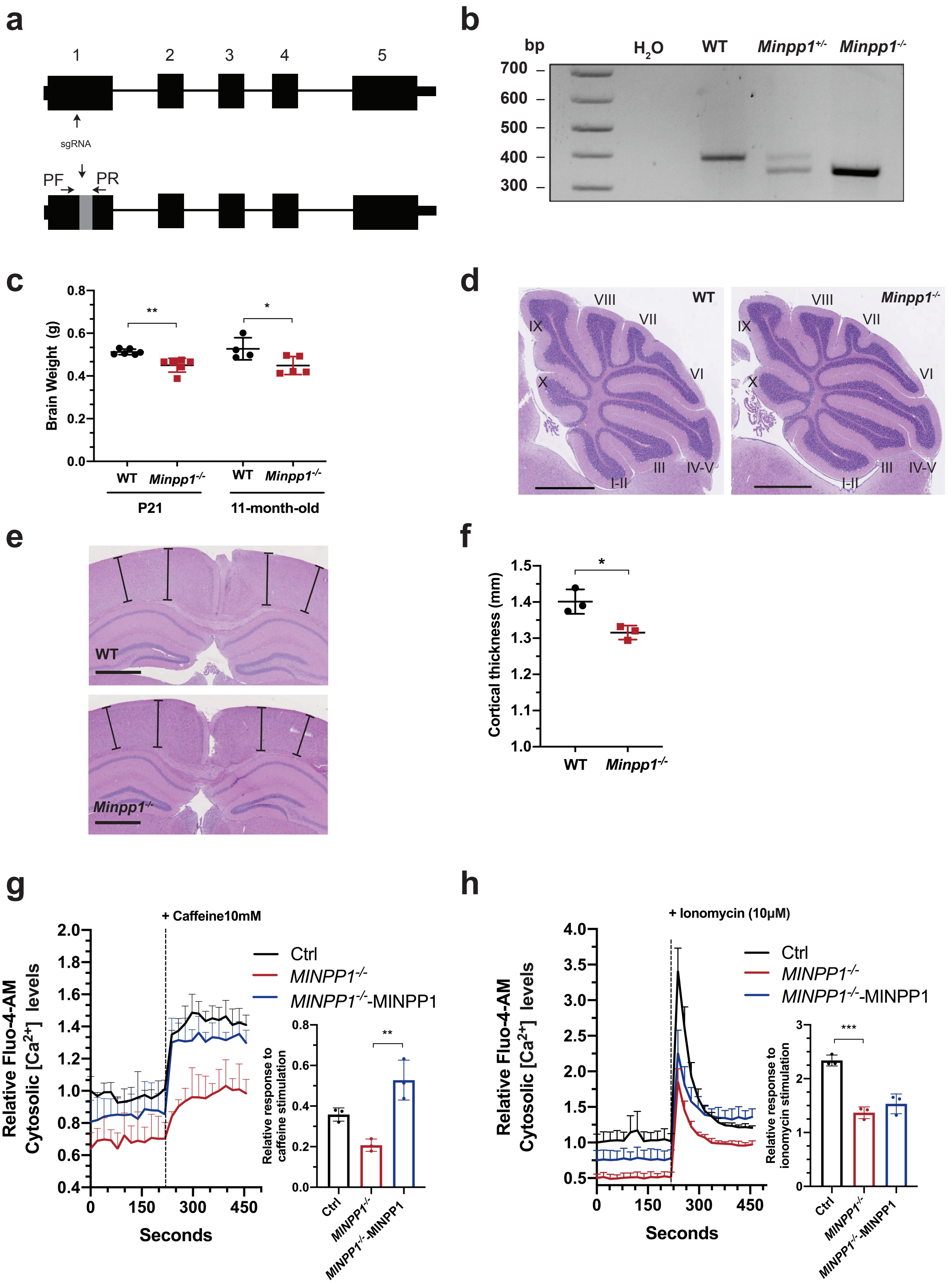

a
1
2
3
4
5
sgRNA
PF
PR
b
bp
H2O
WT
Minpp1+/-
Minpp1-/-
700
600
500
400
300
c
Brain Weight (g)
0.8
0.6
0.4
0.2
0.0
**
*
WT
Minpp1-/-
P21
11-month-old
d
WT
Minpp1-/-
VIII
VII
IX
VI
X
III
IV-V
I-II
e
WT
Minpp1-/-
f
Cortical thickness (mm)
1.5
1.4
1.3
1.2
1.1
1.0
g
+ Caffeine10mM
Relative Fluo-4-AM Cytosolic [Ca2+] levels
Seconds
Ctrl
MINPP1-/-
MINPP1-/--MINPP1
Relative response to caffeine stimulation
h
+ Ionomycin (10μM)
Relative response to ionomycin stimulation
***

**Supplementary Figure 5: Generation and characterization of CRISPR-Cas9 *Minpp1*$^{-/-}$ mouse and $Ca^{2+}$ assays on HEK293 cell lines. (a)** Schema of the sgRNA targeting site and the location of PCR primers. Abbreviations used: Primer Forward (PF) and Primer Reverse (PR). (**b**) PCR based genotyping of *Minpp1*$^{-/-}$ mice indicated successful knockout alleles showing a 31 bp deletion and a smaller PCR product. Uncropped blot is provided as a source data file and representative of six different independent experiments. **(c)** Quantification of total brain weight in grams (g) from P21 and 11-month old WT and *Minpp1*$^{-/-}$ mice. (For P21 N=6, For 11month-old N=4 for control and N=5 for *Minpp1*$^{-/-}$ mice, Two-tailed student's t-test, * and ** indicate p values $p \leq 0.05$ and $p \leq 0.01$, respectively. At P21 Ctrl vs. *Minpp1*$^{-/-}$: p=0.0014; At 11 month-old Ctrl vs. *Minpp1*$^{-/-}$: p=0.0396). (**d**) Representative images of hematoxylin-eosin (HE) staining on P21 sagittal slices of control and mutant cerebellum, scale bar 1 mm. Images are representative of three independent experiments for a total of three mice per genotype. (**e**) Representative images of hematoxylin-eosin (HE) staining on P21 coronal slices of WT and *Minpp1*$^{-/-}$ mutant cortex, scale bar 1 mm. (**f**) Quantification of cortical thickness **(e)** for the WT and *Minpp1*$^{-/-}$ cortex at P21 (N=3 mice, 30 sections per each mouse; Two-tailed Student's t-test, * indicates p value $p \leq 0.05$. Ctrl vs. *Minpp1*$^{-/-}$ p=0.0189). (**g-h**) Relative Fluo-4-AM cytosolic $Ca^{2+}$ levels in control, *MINPP1*$^{-/-}$ HEK293 and MINPP1 over-expression stable *MINPP1*$^{-/-}$ HEK293 cell lines (*MINPP1*$^{-/-}$-MINPP1) loaded either with 10 mM caffeine (**g**) or 10 µM ionomycin **(h)** in the absence of extracellular $Ca^{2+}$. The dotted line indicates the addition of either caffeine (**g**) or ionomycin (**h**). Relative response after caffeine or ionomycin stimulation (peak) is represented graphically (inset). For all of the calcium assay experiments, the data are normalized to cell number with MTT colorimetric assay and presented as mean values relative to control baseline fluorescence intensity control ± s.d. (**g-h**) n=3, one-way ANOVA, Tukey's post hoc test, ** and *** indicate p values $p \leq 0.01$ and $p \leq 0.001$, respectively. For the graphical inset in **(g)**, *MINPP1*$^{-/-}$ vs. *MINPP1*$^{-/-}$-MINPP1: p=0.0018. For

the graphical inset in **(h),** Ctrl vs. *MINPP1*$^{-/-}$: p=0.0003. For **(c)** and **(f-h)**, source data are provided as a source data file.

**Supplementary Table 1. Mendelian Inheritance in the offspring of *Minpp1*$^{+/-}$ × *Minpp1*$^{+/-}$ breedings**

| *Minpp1*$^{+/-}$ × *Minpp1*$^{+/-}$ | Observed (n = 132) | Expected |
|---|---|---|
| WT (*Minpp1*$^{+/+}$), n | 36 | 33 |
| Het (*Minpp1*$^{+/-}$), n | 68 | 66 |
| KO (*Minpp1*$^{-/-}$), n | 28 | 33 |

Expected mendelian inheritance and observed inheritance after *Minpp1*$^{+/-}$ × *Minpp1*$^{+/-}$ breedings, with total observed offspring of 132. p-values of the mendelian ratios are calculated with the Chi-square test and are not significant (ns) (p = 0.57). Abbreviations: WT, wild-type; Het, heterozygous; KO, knock-out.

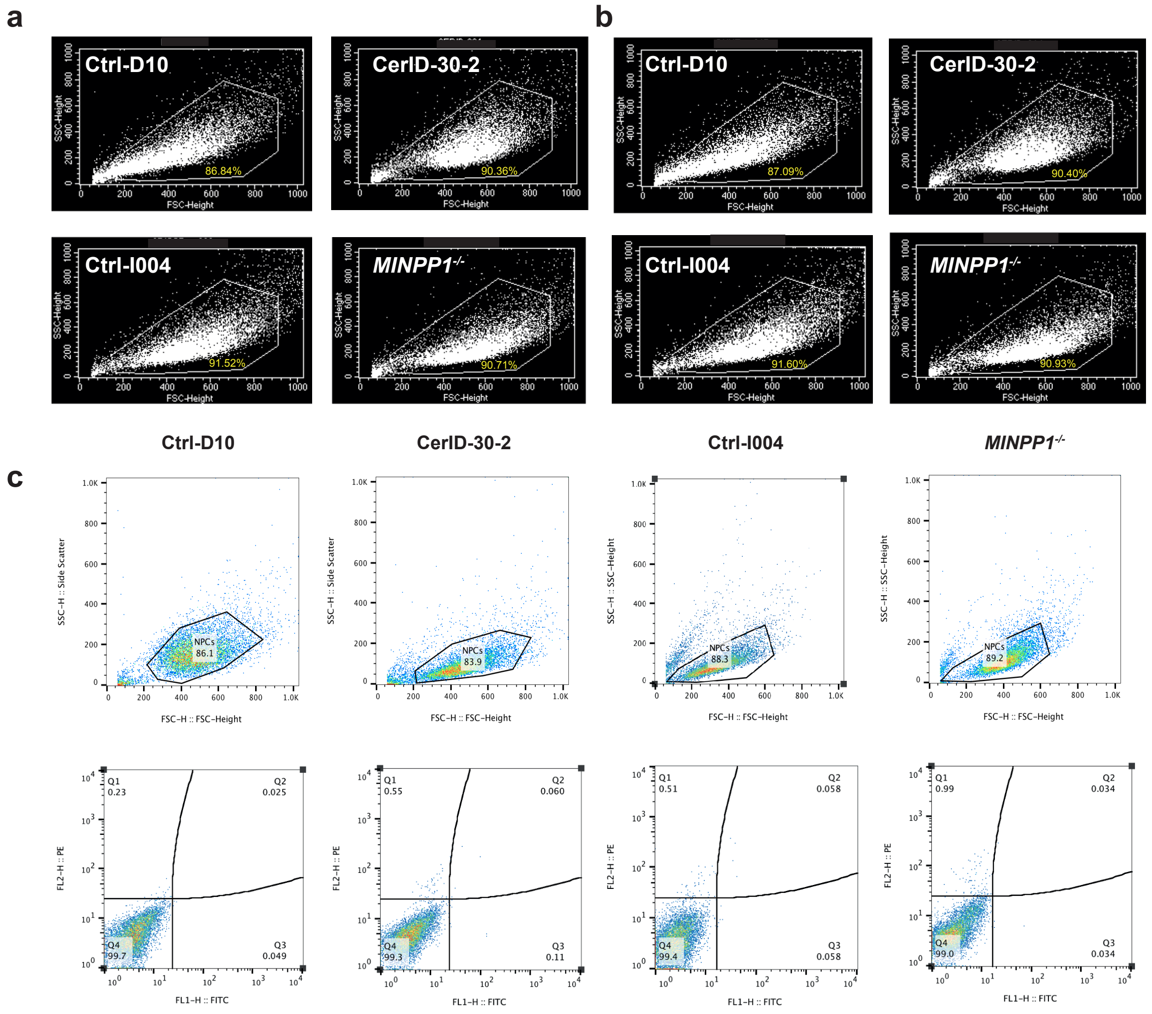

**Supplementary Figure 6. Gating strategy for flow cytometry analysis of iPSCs and their neural derivatives.** Control (Ctrl-D10 and Ctrl-I004), patient-derived (CerID-30-2) and *MINPP1*$^{-/-}$ iPSCs cells stained with SSEA-4 **(a)**, TRA-1-81 **(b)** and their neural derivative counterparts stained with P75 and HNK1 **(c)** are gated based on FSC-H and SSC-H. For the latter cell population, cells that are stained with double negative (FITC and PE) are initially run to set the negative gate (the lower panel in **(c)**).

**Supplementary Table 2. Sequences of primers used in this study**

| Name | Sequence 5'-3' |
|---|---|
| QPCR study | |
| *MINPP1* Forward | TGGACCTCCAACAGTTAATGATAA |
| *MINPP1* Reverse | TGGCACTTGCAAAGTAGCTG |
| *ACTB* Forward | CCCTTGCCATCCTAAAAGCC |
| *ACTB* Reverse | TGCTATCACCTCCCCTGTGT |
| PCR-based genotyping of *Minpp1*$^{-/-}$ mice | |
| *Minpp1* Forward | ACCCTACGACCAAGCAGATC |
| *Minpp1* Reverse | GAGTCCGCAGTCTAGGGAAG |
| Site-directed mutagenesis study | |
| MINPP1.c.157T>G. Forward | GGTTGACATCCTCGTCGCGAGTCTTGGTGCC |
| MINPP1.c.157T>G. Reverse | GGCACCAAGACTCGCGACGAGGATGTCAACC |
| MINPP1.c.1456G>A Forward | AGCAAGGGCTAACAGTACATCTGATAAACTATAAATCTAGAGC |
| MINPP1.c.1456G>A Reverse | GCTCTAGATTTATAGTTTATCAGATGTACTGTTAGCCCTTGCT |

**Supplementary Methods:**

**Construction of human MINPP1 structural model:**

The human MINPP1 structure model was generated with the protein structure homology-modeling server Phyre V2.0 (PMID: 25950237) based on the D. castellii phytase structure (PDB: 2GFI) with 89% sequence coverage. The impact of the missense mutations involving amino-acids included in the MINPP1 structure (i.e. Tyr53, Arg401, Phe228) was evaluated using Missense3D (http://www.sbg.bio.ic.ac.uk/~missense3d/)[1]. All the structural figures were prepared with Pymol (http://www.pymol.org).

**RNA isolation, RT-qPCR and relative fold change expression analysis**

The total RNAs of the different tissues used in this study were ordered from Clontech Laboratories (USA); Human brain (636530); Human fetal brain (636526); Human cerebellum (636535); Human liver (636531); Human lung (636524); Human kidney (636529) and Human skeletal muscle (636534). Total RNA extraction was performed from cell culture pellet using TriZol (ThermoFischer, 15596026) according to the supplier's instructions. The purification of the RNA was done with the RNeasy Mini Kit (Qiagen, 74104). Reverse transcription of the total RNAs was performed using SuperScript II reverse transcriptase (ThermoFisher, 18064022) according to the manufacturer's recommendations. Quantitative PCR was performed with the SYBR Green PCR Master Mix reagent (ThermoFisher, 4364346) on an Applied Biosystems One Step Plus real-time PCR system (Applied Biosystems, 4376600.*ACTB* was chosen as a reference gene to normalize the results between different tissues and cell line. The relative expression of *MINPP1* was determined by the ΔΔCt method using the human brain as the reference tissue.

**Glycosylation Studies :**

The glycosylation status of different MINPP1 mutant forms is checked by following PNGase F protocol according to manufacturer's instructions (P0704L, New England BioLabs).

**Cell Proliferation and Apoptosis Assay with Incucyte-Live-Imaging System**

Cells were seeded on 96-well plate (4379, Essen Bioscience) and cultured with Annexin-V (1:200, 4641, Essen Bioscience) containing media inside the IncuCyte® Live Cell Analysis System S2 (Essen Bioscience). Three images per well were taken with phase contrast and red fluorescence channels every 2 hours for a total of 48 hours, using 20x objective. Images were then analysed with Incucyte-Zoom software 2016A, by defining a mask with the basic analyzer. The same mask was applied to all time points.

**Differentiation of iPSCs towards dorsal telencephalic progenitors**

iPSCs were differentiated towards dorsal telencephalic lineage[2] . Briefly, iPSCs were manually dissociated into big clumps and floated in non-coated dishes in neural induction media N2B27 (1:1 mix of DMEM-F12 (31331093, Gibco) and Neural Basal Medium (1103049, Gibco)) supplemented with N2 (07156, STEMCELL), NeuroCult SM1 without vitamin-A (05731, STEMCELL), bFGF (10 ng/ml, PHG0264, Invitrogen) and dual-SMAD inhibition small molecules SB431542 (20 µM, 72232, STEMCELL) and LDN193189 (HCl) (500 nM, 72146,

STEMCELL). After 6 hours, embryoid body (EB)-like structures were seeded on polyornithine (0.1 mg/ml, P4957, Sigma) and laminin (4 µg/ml) pre-coated 3.5 cm dishes. The media were changed every 3 days. At day 12-15, the neural rosettes were manually or enzymatically passaged on to polyornithine-laminin-pre-coated dishes and cultured in N2B27 medium supplemented with bFGF (10 ng/ml), EGF (10 ng/ml, PHG0314, Invitrogen) and BDNF (20 ng/ml, 78005, STEMCELL). At confluence, cells were passaged with a density of $5x10^4/cm^2$ and the media was supplemented with Y-27632 (10µM) on the day of passage. Neuroectodermal origin of the emerging neural progenitor-like cells was assessed by HNK1/P75 Flow cytometry (Supplementary Fig.3h, Supplementary Fig. 6c).

**BrdU Proliferation Assay**

Initially, the cells were incubated with 10 µM BrdU labelling solution (B5002-1G, Sigma) for 6 hours at 37 °C in a $CO_2$ incubator. The cells were further thoroughly washed thrice with PBS and fixed with 4% paraformaldehyde for 15 minutes. Permeabilization of the cells were performed using 0.25% PBS-Triton-X-100 for 10 minutes. After 3 washes with PBS, DNA hydrolysis was achieved by incubating the cells with 2 M HCL for 30 minutes at 37 °C. Further, the cells were neutralized with 0.1 M sodium borate buffer (pH 8.5) for 30 minutes at room temperature. Cells were thoroughly washed with PBS and immunostaining of the cells with BrdU antibody (1:250, ab6326, Abcam) was carried out as previously mentioned in the immunofluorescence section.

**Flow Cytometry**

Cells were fixed with 1% paraformaldehyde and cell suspensions in PBS were stained for 30 minutes with anti-SSEA-4-FITC (Miltenyi biotec 130–098-371), anti-TRA1-81-FITC (BD Biosciences, 560194), anti-HNK1-FITC (130-092-174, MiltenyiBiotec), anti-P75-APC (130-110-079, MiltenyiBiotec), REA control-FITC (130-113-449, MiltenyiBiotec), or REA Control -PE (130-113-450, MiltenyiBiotec) at 1/10 dilution. Prior to analysis, the samples were centrifuged and resuspended in PBS. The samples were then processed with FACSCalibur (BD Biosciences) and analyzed with FlowJo Software 10.3 and BD CellQuest Pro software 5.1.

***In vitro* phytase Assay**

The *in vitro* phytase assay was performed as previously described[4]. Briefly, 4 nmol of $IP_6$ was added to 1 ml of conditioned media from Ctrl and *MINPP1*$^{-/-}$ HEK293 cells and further

incubated for 0, 2, 4 and 6 hours at 37 °C. The samples were then mixed with Orange G loading dye and resolved by 35% PAGE followed by toluidine blue staining.

**Analysis of extracellular inositol phosphates**

For analysis of extracellular inositol phosphates, cells were labelled for 5 days with [3H]-inositol as indicated above. Media were collected and centrifuged at 300xg for 5 minutes to remove cell contaminants. Perchloric acid was added to the supernatant to final 1 M. The acidified medium was incubated on ice for 10 minutes before centrifugation at 18,000 xg to remove precipitates. Inositol phosphates in the supernatant were then purified using titanium dioxide beads[5]. Briefly, 4 mg beads were added to the media extracts and the samples rotated at 4°C for 15 minutes. Beads were washed twice in 1 M perchloric acid, before elution with ammonium hydroxide. Radiolabeled inositol phosphates in the purified media extracts were separated by SAX-HPLC as before, and radioactivity in each fraction measured with a beta counter.

**Supplementary Note:**

**Detailed method for Sequencing, Variant Filtering and Prioritization**

**Families CerID-09, 11 and 30**

Genomic DNA was extracted from peripheral blood cells. As previously published [6] [7], exome capture was performed with the the SureSelect Human All Exon kit (Agilent Technologies). Agilent Sure Select Human All Exon (54Mb Clinical Research Exome) libraries were prepared from 3µg of genomic DNA sheared with an Ultrasonicator (Covaris) as recommended by the manufacturer. Barcoded exome libraries were pooled and sequenced with a HiSeq2500 system (Illumina), generating paired-end reads. The mean depth of coverage of the exome libraries was greater than ~100-120X with >98 to 99% of the targeted exonic bases covered at least by 15 independent reads and >93 to 98% by at least 30 independent sequencing reads (98-99% at 15X and 93 to 98% at 30X). After demultiplexing, sequences were aligned to the reference human genome hg19 using the Burrows-Wheeler Aligner (BWA). Downstream processing was carried out with the Genome Analysis Toolkit (GATK), SAMtools and Picard, following documented best practices (http://www.broadinstitute.org/gatk/guide/topic?name=best-practices). Variant calls were made with the GATK HaplotypeCaller (V3.7). The annotation process was based on the latest release of the Ensembl database, Gnomad 2.1. Variants were annotated and analyzed using the Polyweb software interface designed by the Bioinformatics platform of University Paris Descartes. Variants were filtered to less than 1% minor allele frequency in population databases and with MAF <0.001 in our sequenced cohort that include more than 15000 exomes. Manual curation of the known PCH genes (for the gene list see [6] ) was first performed to exclude

a known genetic cause. Variants were then prioritized by the suspected mode of inheritance, OMIM status and *in silico* prediction tools Mutation Taster [8], SIFT, Polyphen [9] and CADD score [10]. Candidate genes were correlated to the patient's phenotype, gene function and expression. Sanger sequencing of the patient and her parents was performed to confirm the variants and co-segregation using a 3500xl genetic analyzer (4405633; Applied Biosystems). For the family CerID-09, homozygous variants were identified in 31 genes. From these analyses, 3 genes with the predicted most likely pathogenic variants (*MINPP1*; *ZCCHC24* and *NKX1-2*) were further considered as candidates. For the family CerID-11, 4 variants were identified in 2 potential candidate genes with 2 heterozygous variants (*MINNP1* ; *KIAA1522*). For the family CerID-30, 21 variants were identified in 11 potential candidate genes including 6 genes with 2 or more heterozygous variants, and 5 genes with one homozygous variant. Two genes with most likely pathogenic variants were further explored: *MINPP1* and *MTFMT*. The involvement of MTFMT was excluded based on the absence of clinical correlations.

**Family TR-PCH-01**

Patient TR-PCH-01-1 was part of a replication cohort of 100 patients with congenital or early-onset cerebellar defects that includes 21 PCH cases. This cohort was investigated using a targeted gene panel. This panel includes 184 known genes involved in congenital cerebellar anomalies (list available upon request), and 13 candidate genes including *MINPP1*. Samples were prepared using Blood DNA and the SeqCap EZ Choice preparation kit (Roche) and sequenced on an Illumina MiSeq sequencer using 2 × 150 bp sequencing kits. The Basespace cloud computing platform (with BWA 2.1 and GATK Unified Genotyper 1.6) and the Variant Studio v.3.0 software provided by Illumina were used. Other known PCH genes were excluded for TR-PCH1-1. Pathogenicity of variants was ascertained according to the ACMG (American College of Medical Genetics) criteria. The segregation was confirmed by Sanger sequencing in the index case, both parents and unaffected sibling.

**Families PCH-2456 and PCH-2712**

Blood DNA was extracted using Qiagen reagents (Qiagen Inc., USA) and whole exome sequencing was performed on all affected family members using the Illumina HiSeq2500 which yields 100 bp paired-end reads covering 80% of the exome at 20X. GATK best practices pipeline was used for SNP and INDEL variant identification. Variants were prioritized by allele frequency (<0.1% MAF in our exome database of over 5000 individuals), conservation and predicted effect on protein function. The variants were predicted to be disease causing and were absent from 1000 Genomes, ExAC and gnomAD. Sanger sequencing confirmed segregation according to a strict recessive mode of inheritance in all genetically informative and available family members.

**REFERENCES**

1. Ittisoponpisan, S. *et al.* Can Predicted Protein 3D Structures Provide Reliable Insights into whether Missense Variants Are Disease Associated? *J Mol Biol* **431**, 2197-2212 (2019).
2. Boissart, C. *et al.* Differentiation from human pluripotent stem cells of cortical neurons of the superficial layers amenable to psychiatric disease modeling and high-throughput drug screening. *Transl Psychiatry* **3**, e294 (2013).

3. Medina-Cano, D. *et al.* High N-glycan multiplicity is critical for neuronal adhesion and sensitizes the developing cerebellum to N-glycosylation defect. *Elife* **7**(2018).
4. Wilson, M.S., Bulley, S.J., Pisani, F., Irvine, R.F. & Saiardi, A. A novel method for the purification of inositol phosphates from biological samples reveals that no phytate is present in human plasma or urine. *Open Biol* **5**, 150014 (2015).
5. Wilson, M.S. & Saiardi, A. Inositol Phosphates Purification Using Titanium Dioxide Beads. *Bio Protoc* **8**(2018).
6. Chemin, J. *et al.* De novo mutation screening in childhood-onset cerebellar atrophy identifies gain-of-function mutations in the CACNA1G calcium channel gene. *Brain* **141**, 1998-2013 (2018).
7. Megahed, H. *et al.* Utility of whole exome sequencing for the early diagnosis of pediatric-onset cerebellar atrophy associated with developmental delay in an inbred population. *Orphanet J Rare Dis* **11**, 57 (2016).
8. Schwarz, J.M., Cooper, D.N., Schuelke, M. & Seelow, D. MutationTaster2: mutation prediction for the deep-sequencing age. *Nat Methods* **11**, 361-2 (2014).
9. McLaren, W. *et al.* The Ensembl Variant Effect Predictor. *Genome Biol* **17**, 122 (2016).
10. Rentzsch, P., Witten, D., Cooper, G.M., Shendure, J. & Kircher, M. CADD: predicting the deleteriousness of variants throughout the human genome. *Nucleic Acids Res* **47**, D886-D894 (2019).
