# Supplementary material for: MINPP1 prevents intracellular accumulation of the chelator inositol hexakisphosphate and is mutated in Pontocerebellar Hypoplasia
Source: Nat Commun. 2020 Nov 30;11:6087. doi: 10.1038/s41467-020-19919-y (PMC7705663; doi:10.1038/s41467-020-19919-y)
Supplement: Supplementary file 3 — Reporting Summary [file 41467_2020_19919_MOESM3_ESM.pdf]

nature research

Corresponding author(s): Vincent Cantagrel and Adolfo Saiardi

Last updated by author(s): 2020/09/29

Reporting Summary

Nature Research wishes to improve the reproducibility of the work that we publish. This form provides structure for consistency and transparency in reporting. For further information on Nature Research policies, see Authors & Referees and the Editorial Policy Checklist .

Statistics

For all statistical analyses, confirm that the following items are present in the figure legend, table legend, main text, or Methods section.

n/a	Confirmed	
☐	☒	The exact sample size (n) for each experimental group/condition, given as a discrete number and unit of measurement
☐	☒	A statement on whether measurements were taken from distinct samples or whether the same sample was measured repeatedly
☐	☒	The statistical test(s) used AND whether they are one- or two-sided<br>*Only common tests should be described solely by name; describe more complex techniques in the Methods section.*
☒	☐	A description of all covariates tested
☐	☒	A description of any assumptions or corrections, such as tests of normality and adjustment for multiple comparisons
☐	☒	A full description of the statistical parameters including central tendency (e.g. means) or other basic estimates (e.g. regression coefficient) AND variation (e.g. standard deviation) or associated estimates of uncertainty (e.g. confidence intervals)
☐	☒	For null hypothesis testing, the test statistic (e.g. F, t, r) with confidence intervals, effect sizes, degrees of freedom and P value noted<br>*Give P values as exact values whenever suitable.*
☒	☐	For Bayesian analysis, information on the choice of priors and Markov chain Monte Carlo settings
☒	☐	For hierarchical and complex designs, identification of the appropriate level for tests and full reporting of outcomes
☒	☐	Estimates of effect sizes (e.g. Cohen's d, Pearson's r), indicating how they were calculated

Our web collection on statistics for biologists contains articles on many of the points above.

Software and code

Policy information about availability of computer code

Data collection	Polyweb, Image Studio Lite Ver 5.2, Incucyte Zoom software 2016A
Data analysis	ImageJ Java 1.8.0 ,GraphPad Prism8, Microsoft Excel 2016, NDP.view.2, FlowJo Software 10.3, BD CellQuest Pro software 5.1 , COBALT: Multiple Alignment tool

For manuscripts utilizing custom algorithms or software that are central to the research but not yet described in published literature, software must be made available to editors/reviewers. We strongly encourage code deposition in a community repository (e.g. GitHub). See the Nature Research guidelines for submitting code & software for further information.

Data

Policy information about availability of data

All manuscripts must include a data availability statement. This statement should provide the following information, where applicable:

- Accession codes, unique identifiers, or web links for publicly available datasets
- A list of figures that have associated raw data
- A description of any restrictions on data availability

Source data for the HPLC results and uncropped blots are provided with this paper. The other datasets generated and/or analysed during the current study are available from the corresponding author on reasonable request

Field-specific reporting

Please select the one below that is the best fit for your research. If you are not sure, read the appropriate sections before making your selection.

Life sciences study design

All studies must disclose on these points even when the disclosure is negative.

Sample size	Sample size was as large as possible with at least three independent replicates in critical experiments and the number was sufficient to support the statistical analyses performed in this manuscripts. For mouse experiments, we achieved > 3 mice per genotype per experiment and/or condition to reach statistical significance with the minimum number of animals.<br>All available human samples i.e., fibroblasts or iPSC line from patients, and the sample size was not pre-determined.
Data exclusions	No data were excluded except MTT based assay (Fig.2 D, 5 E-H), where outliners are significantly detected with Graphpad outliners and are excluded (https://www.graphpad.com/quickcalcs/Grubbs1.cfm). This pre-established automatic detection of outliers was applied to an experiment based a large number of samples with multiple processing steps.
Replication	All attempts of replication were succesfull. The number of independent biologic replicates is indicated in each Figure Legend.
Randomization	In vitro samples and Mouse samples were allocated based on genotype.
Blinding	For animal studies, brain weight and cortical thickness were assesed by blinding the investigator to the genotype. Immunofluorescent staining-based cell counting was performed without the knowledge of the group allocation.

Behavioural & social sciences study design

All studies must disclose on these points even when the disclosure is negative.

Study description	*Briefly describe the study type including whether data are quantitative, qualitative, or mixed-methods (e.g. qualitative cross-sectional, quantitative experimental, mixed-methods case study).*
Research sample	*State the research sample (e.g. Harvard university undergraduates, villagers in rural India) and provide relevant demographic information (e.g. age, sex) and indicate whether the sample is representative. Provide a rationale for the study sample chosen. For studies involving existing datasets, please describe the dataset and source.*
Sampling strategy	*Describe the sampling procedure (e.g. random, snowball, stratified, convenience). Describe the statistical methods that were used to predetermine sample size OR if no sample-size calculation was performed, describe how sample sizes were chosen and provide a rationale for why these sample sizes are sufficient. For qualitative data, please indicate whether data saturation was considered, and what criteria were used to decide that no further sampling was needed.*
Data collection	*Provide details about the data collection procedure, including the instruments or devices used to record the data (e.g. pen and paper, computer, eye tracker, video or audio equipment) whether anyone was present besides the participant(s) and the researcher, and whether the researcher was blind to experimental condition and/or the study hypothesis during data collection.*
Timing	*Indicate the start and stop dates of data collection. If there is a gap between collection periods, state the dates for each sample cohort.*
Data exclusions	*If no data were excluded from the analyses, state so OR if data were excluded, provide the exact number of exclusions and the rationale behind them, indicating whether exclusion criteria were pre-established.*
Non-participation	*State how many participants dropped out/declined participation and the reason(s) given OR provide response rate OR state that no participants dropped out/declined participation.*
Randomization	*If participants were not allocated into experimental groups, state so OR describe how participants were allocated to groups, and if allocation was not random, describe how covariates were controlled.*

Ecological, evolutionary & environmental sciences study design

All studies must disclose on these points even when the disclosure is negative.

Study description	*Briefly describe the study. For quantitative data include treatment factors and interactions, design structure (e.g. factorial, nested, hierarchical), nature and number of experimental units and replicates.*
Research sample	*Describe the research sample (e.g. a group of tagged Passer domesticus, all Stenocereus thurberi within Organ Pipe Cactus National Monument), and provide a rationale for the sample choice. When relevant, describe the organism taxa, source, sex, age range and any manipulations. State what population the sample is meant to represent when applicable. For studies involving existing datasets, describe the data and its source.*
Sampling strategy	*Note the sampling procedure. Describe the statistical methods that were used to predetermine sample size OR if no sample-size calculation was performed, describe how sample sizes were chosen and provide a rationale for why these sample sizes are sufficient.*

Data collection	*Describe the data collection procedure, including who recorded the data and how.*
Timing and spatial scale	*Indicate the start and stop dates of data collection, noting the frequency and periodicity of sampling and providing a rationale for these choices. If there is a gap between collection periods, state the dates for each sample cohort. Specify the spatial scale from which the data are taken*
Data exclusions	*If no data were excluded from the analyses, state so OR if data were excluded, describe the exclusions and the rationale behind them, indicating whether exclusion criteria were pre-established.*
Reproducibility	*Describe the measures taken to verify the reproducibility of experimental findings. For each experiment, note whether any attempts to repeat the experiment failed OR state that all attempts to repeat the experiment were successful.*
Randomization	*Describe how samples/organisms/participants were allocated into groups. If allocation was not random, describe how covariates were controlled. If this is not relevant to your study, explain why.*
Blinding	*Describe the extent of blinding used during data acquisition and analysis. If blinding was not possible, describe why OR explain why blinding was not relevant to your study.*

Did the study involve field work? ☐ Yes ☐ No

Field work, collection and transport

Field conditions	*Describe the study conditions for field work, providing relevant parameters (e.g. temperature, rainfall).*
Location	*State the location of the sampling or experiment, providing relevant parameters (e.g. latitude and longitude, elevation, water depth).*
Access and import/export	*Describe the efforts you have made to access habitats and to collect and import/export your samples in a responsible manner and in compliance with local, national and international laws, noting any permits that were obtained (give the name of the issuing authority, the date of issue, and any identifying information).*
Disturbance	*Describe any disturbance caused by the study and how it was minimized.*

Reporting for specific materials, systems and methods

We require information from authors about some types of materials, experimental systems and methods used in many studies. Here, indicate whether each material, system or method listed is relevant to your study. If you are not sure if a list item applies to your research, read the appropriate section before selecting a response.

Materials & experimental systems

n/a	Involved in the study	
☐	☒	Antibodies
☐	☒	Eukaryotic cell lines
☒	☐	Palaeontology
☐	☒	Animals and other organisms
☐	☒	Human research participants
☒	☐	Clinical data

Methods

n/a	Involved in the study	
☒	☐	ChIP-seq
☐	☒	Flow cytometry
☒	☐	MRI-based neuroimaging

Antibodies

Antibodies used	Anti-PAX6(901301, BioLegend), Anti-TUBULIN β-3 (801201, BioLegend), Anti-OCT4 (sc-5279, Santa-Cruz), Anti-SOX2 (AB5603, Millipore), Anti-MINPP1 (sc-10399, Santa-Cruz), Anti-β Actin (AM4302, Invitrogen), anti BrdU antibody (ab6326, Abcam), anti-NESTIN (1:200,N5413, Sigma), anti-HNK1-FITC (130-092-174, MiltenyiBiotec), anti-P75-PE (130-110-079, MiltenyiBiotec) REA control-FITC (130-113-449, MiltenyiBiotec), anti-SSEA-4-FITC (Miltenyi biotec 130–098-371), anti-TRA1-81-FITC (BD Biosciences, 560194), REA Control -PE (130-113-450,MiltenyiBiotec), Alexa-Fluor-coupled secondary antibodies (A21424, A-11034, Invitrogen), IRDye-coupled secondary antibodies (925-68070, 925-32211, LICOR) .
Validation	Validation of MINPP1 is provided in the manuscript and in PMID: 23186306. For Anti-OCT4,ANTI-SOX2 and Anti-β Actin (AM4302, Invitrogen): PubMed PMID: 30311906; For Anti-PAX6(901301, BioLegend), Anti-TUBULIN β-3 (801201, BioLegend) : PubMed PMID: 31006631; Anti-Calmodulin (465, Swant): PubMed PMID: 6163780; anti-NESTIN (1:200,N5413, Sigma) Pubmed PMID 23508200; anti-HNK1-FITC (130-092-174, MiltenyiBiotec): PubMed PMID: 16339584; anti-P75-PE (130-110-079, MiltenyiBiotec): PubMed PMID:20367498; anti-SSEA-4-FITC (Miltenyi biotec 130–098-371) :PubMed PMID: 26607327; anti-TRA1-81-FITC (BD Biosciences, 560194) PUBMED PMID: 9804556.

Eukaryotic cell lines

Policy information about cell lines

Cell line source(s)	hiPSCs are generated by Imagine Institute's iPSC platform and Duke iPSC Share Resource Facility. Patient fibroblasts were obtained from skin biopsies. HEK293T cells were from ATCC (Teddington, Middlesex, United Kingdom).
Authentication	hiPSCs used are provided from Imagine Institute's iPSC platform and Duke iPSC Share Resource Facility. No unusual chromosomal abnormalities are detected by CGH array (60K) in all of the hiPSCs lines.
Mycoplasma contamination	All cell lines tested are negative for mycoplasma contamination.
Commonly misidentified lines (See ICLAC register)	No commonly misidentified cell lines were used.

Palaeontology

Specimen provenance	*Provide provenance information for specimens and describe permits that were obtained for the work (including the name of the issuing authority, the date of issue, and any identifying information).*
Specimen deposition	*Indicate where the specimens have been deposited to permit free access by other researchers.*
Dating methods	*If new dates are provided, describe how they were obtained (e.g. collection, storage, sample pretreatment and measurement), where they were obtained (i.e. lab name), the calibration program and the protocol for quality assurance OR state that no new dates are provided.*

☐ Tick this box to confirm that the raw and calibrated dates are available in the paper or in Supplementary Information.

Animals and other organisms

Policy information about studies involving animals; ARRIVE guidelines recommended for reporting animal research

Laboratory animals	The genetic background used to generate the Minppp1 mutant mouse is C57BL/6J. Both males and females were used at age postnatal day 21 (P21) and at embryonic day 14 (E14.5). Standard housing and experimental conditions were used , all approved by the Paris Descartes University ethical comity and the MESR.
Wild animals	No wild animals were used in the study
Field-collected samples	No field collected samples were used in this study
Ethics oversight	In this study, animals were used in compliance with the French Animal Care and Use Committee from the Paris Descartes University (APAFIS#961-201506231137361).

Note that full information on the approval of the study protocol must also be provided in the manuscript.

Human research participants

Policy information about studies involving human research participants

Population characteristics	All informations related to participants are included in table 2 and Supplementary Note.
Recruitment	The families included in this study were referred to the Necker Hospital genetic and neurology departments and to collaborative centers.
Ethics oversight	The collection of samples and research investigations related to human participants has been approved by the French ministry (MESR) and the ethical comity of the Imagine Institute.

Note that full information on the approval of the study protocol must also be provided in the manuscript.

ChIP-seq

Data deposition

☐ Confirm that both raw and final processed data have been deposited in a public database such as GEO.

☐ Confirm that you have deposited or provided access to graph files (e.g. BED files) for the called peaks.

Data access links<br>*May remain private before publication.*	*For "Initial submission" or "Revised version" documents, provide reviewer access links. For your "Final submission" document, provide a link to the deposited data.*
Files in database submission	*Provide a list of all files available in the database submission.*

Genome browser session
(e.g. UCSC)

Provide a link to an anonymized genome browser session for "Initial submission" and "Revised version" documents only, to enable peer review. Write "no longer applicable" for "Final submission" documents.

Methodology

Replicates

Describe the experimental replicates, specifying number, type and replicate agreement.

Sequencing depth

Describe the sequencing depth for each experiment, providing the total number of reads, uniquely mapped reads, length of reads and whether they were paired- or single-end.

Antibodies

Describe the antibodies used for the ChIP-seq experiments; as applicable, provide supplier name, catalog number, clone name, and lot number.

Peak calling parameters

Specify the command line program and parameters used for read mapping and peak calling, including the ChIP, control and index files used.

Data quality

Describe the methods used to ensure data quality in full detail, including how many peaks are at FDR 5% and above 5-fold enrichment.

Software

Describe the software used to collect and analyze the ChIP-seq data. For custom code that has been deposited into a community repository, provide accession details.

Flow Cytometry

Plots

Confirm that:

- [x] The axis labels state the marker and fluorochrome used (e.g. CD4-FITC).
- [x] The axis scales are clearly visible. Include numbers along axes only for bottom left plot of group (a 'group' is an analysis of identical markers).
- [x] All plots are contour plots with outliers or pseudocolor plots.
- [x] A numerical value for number of cells or percentage (with statistics) is provided.

Methodology

Sample preparation

Neural rosette cultures differentiated from hiPSCs were fixed with 1% paraformaldehyde and cell suspensions in PBS were stained for 30 minutes with the appropriate markers. Prior to analysis, the samples were centrifuged and resuspended in PBS.

Instrument

FACS Calibur (BD Biosciences)

Software

FlowJO Software (Treestar, Ashland, OR)

Cell population abundance

The cell population is initially gated according to FSC and SSC in order to identify cells of interest based on size and granularity. The FSC/SSC set up is done according to Imagine Institute iPSCs core facilities set up. Cell population abundance are recorded with the samples that are stained with double positive markers (HNK1-FITC, P75-PE, SSEA4 FITC, TRA 1-81 FITC) and the percentage of the relevant cell populations are marked on the figure.

Gating strategy

Cells that were stained with double negative (FITC and PE) were initially run in order to allow the cell population appear on the middle of the 3th quadrant. Then, cells that were singly-stained with either (HNK1-FITC, P75-PE, SSEA4-FITC, TRA 1-81-FITC) were run to perform compensate corrections for the PE and FITC channel respectively. Once the set-up is finished, the double stained samples were passed and the data is recorded.

- [x] Tick this box to confirm that a figure exemplifying the gating strategy is provided in the Supplementary Information.
